# Oxidative damage to macromolecules in the thyroid - experimental evidence

**DOI:** 10.1186/1756-6614-5-25

**Published:** 2012-12-27

**Authors:** Małgorzata Karbownik-Lewińska, Agnieszka Kokoszko-Bilska

**Affiliations:** 1 Department of Oncological Endocrinology, Medical University of Lodz, 7/9 Zeligowski St, Lodz, 90-752, Poland; 2 Department of Endocrinology and Metabolic Diseases, Polish Mother’s Memorial Hospital – Research Institute, 281/289 Rzgowska St, Lodz, 93-338, Poland

**Keywords:** The thyroid gland, Thyroid hormone synthesis, Oxidative stress, Free radical, Antioxidant, Prooxidant, Hydrogen peroxide, Iron

## Abstract

Whereas oxidative reactions occur in all tissues and organs, the thyroid gland constitutes such an organ, in which oxidative processes are indispensable for thyroid hormone synthesis. It is estimated that huge amount of reactive oxygen species, especially of hydrogen peroxide (H_2_O_2_), are produced in the thyroid under physiological conditions, justifying the statement that the thyroid gland is an organ of “oxidative nature”. Apart from H_2_O_2_, also other free radicals or reactive species, formed from iodine or tyrosine residues, participate in thyroid hormone synthesis. Under physiological conditions, there is a balance between generation and detoxification of free radicals. Effective protective mechanisms, comprising antioxidative molecules and the process of compartmentalization of potentially toxic molecules, must have been developed in the thyroid to maintain this balance. However, with additional oxidative abuse caused by exogenous or endogenous prooxidants (ionizing radiation being the most spectacular), increased damage to macromolecules occurs, potentially leading to different thyroid diseases, cancer included.

## Introduction

Reactive oxygen species (ROS) and free radicals participate in numerous metabolic processes. Under physiological conditions, there is a balance between the production and detoxification of ROS [1,2]. However, any internal or external pathological factor may disrupt this balance, leading to conditions referred to as oxidative stress, playing a significant role in the pathogenesis of several diseases [1].

The most basic reaction of oxidative stress is Fenton reaction:

(1) $$Fe^{2+}+H_{2}O_{2}\to Fe^{3+}{+}^{•}\;OH+OH^{-}$$

The most harmful free radical is hydroxyl radical (^•^OH), which is produced during this reaction. Both substrates of Fenton reaction are normally present in cells and possess important physiological roles.

Iron is an essential element for normal metabolic processes, being a cofactor for many biological reactions. On the other hand, free ionic iron, as a potent generator of ROS, can enhance oxidative stress. It is known that increased iron stores in tissue result in cellular toxicity and are associated with increased risk of cellular damage, cancer initiation included [1,3,4].

Hydrogen peroxide (H_2_O_2_) is a form of ROS. All cells produce continuously low or moderate amounts of H_2_O_2_ under physiological conditions. This ROS is generated mainly by the intracellular family of NADPH oxidases (NOX). At concentrations higher than physiological, H_2_O_2_ enhances oxidative stress, leading to increased DNA oxidative damage and consequent mutagenesis [5,6].

Bivalent iron (ferrous ion; Fe^2+^) and/or H_2_O_2_, which initiate Fenton reaction are frequently used to experimentally induce oxidative damage to macromolecules [7-14].

Whereas oxidative reactions occur in all tissues and organs, the thyroid gland constitutes such an organ, in which oxidative processes are indispensable for thyroid hormone synthesis. It is estimated that huge amount of ROS, especially of H_2_O_2_, are produced in the thyroid under physiological conditions, justifying the statement that the thyroid gland is an organ of “oxidative nature”.

Hydrogen peroxide is an essential factor for thyroid hormone biosynthesis. It is produced in the thyroid gland by two isoform enzymes, dual oxidase 1 (DUOX1) and 2 (DUOX2), belonging to NOX family, with the most convincing experimental evidence found for DUOX2 [15,16]. Both DUOX enzymes are expressed in the apical plasma membrane of thyroid follicular cells (thyrocytes) [15,16]. In turn, NOX4 playing also an important role in the synthesis of H_2_O_2_ in the thyroid, acts intracellulary [17]. Hydrogen peroxide acts as an electron acceptor at each step of thyroid hormone synthesis, namely at iodide oxidation and, next, at its organification, as well as at coupling reaction of iodotyrosines [18]. It is essential for activity of thyroperoxidase (TPO) – the key enzyme for thyroid hormone synthesis.

Thyroperoxidase is a heme-dependent protein, thus it contains iron atoms in its structure. Hydrogen peroxide participates in autocatalytic covalent heme binding to the apoprotein of TPO molecule, thereby stabilizing the activity of the enzyme [19]. Hydrogen peroxide availability is the rate-limiting step in thyroid hormone biosynthesis, although H_2_O_2_ is produced in large excess compared with the amount of iodide incorporated into proteins. This may be due to relatively high Michaelis-Menten constant of TPO for H_2_O_2_, which means that relatively high concentrations of H_2_O_2_, as a substrate, are required to properly activate the enzyme [20,21]. Large quantities and membrane permeable nature of H_2_O_2_ can lead to its diffusion from the luminal side of the apical membrane back to the cell. Because iron is present in TPO and H_2_O_2_ is indispensable for TPO activity, the thyroid gland may be exposed – under certain conditions – to excessive amounts of either Fe^2+^ or H_2_O_2_, or both, creating favorable conditions for additional Fenton reaction and, consequently, oxidative damage.

Thyrotropin (TSH), the main secretory and growth stimulatory factor for the thyroid, is obviously involved in the production of H_2_O_2_ in that gland. The major meaning of that fact is that an increased production of H_2_O_2_, with subsequently enhanced formation of free radicals (especially ^•^OH), takes place in any conditions accompanied by the increased blood TSH concentration. Thus, TSH stimulation results in goitre formation and, under certain conditions, in thyroid cancer initiation via the mechanism of, at least in part, oxidative stress. In agreement with this, oxidative stress creates required conditions for thyroid cell proliferation [22,23].

Apart from H_2_O_2_, numerous other reactive species are formed in the thyroid, such as, for example, nitric oxide (NO^•^). Also particular elements of thyroid hormone synthesis pathway are partially confirmed to exist as free radicals or other reactive species, such as: tyrosine free radical (Tyr^**•**^), diiodotyrosyl residue radical (DIT^•^), diiodotyrosyl residue radical in thyroglobulin (Tg-DIT^•^), iodine radical (I^•^), iodinium ion (I^+^), hypoiodous acid intermediate [IO^-^ (IOH)], and ascorbate radical (Asc^•^) [24].

Due to potential huge oxidative stress in the thyroid gland, effective protective mechanisms should have been developed. An antioxidative defence system in the thyroid gland comprises both antioxidative enzymes and free radical scavengers.

The presence of the following antioxidative enzymes in the thyroid gland has been documented: superoxide dismutase (SOD), glutathione (GSH) peroxidase (GSH-Px) and catalase (CAT) [25]. In turn, antioxidants such as α- and γ-tocopherols, coenzyme Q, and ascorbic acid have also been found in the thyroids of different species. Among antioxidants present in the thyroid, peroxiredoxins (Prxs) seem to be of a special value, as they are involved in the process of H_2_O_2_ elimination, when this oxygen species is formed in response to TSH, and they protect thyroid cells from H_2_O_2_-induced apoptosis [25,26].

The results, obtained in humans and in animal models, suggest that oxidative damage in the thyroid, especially this associated with thyroid cancer, is accompanied by increased activities of antioxidative enzymes or increased production of antioxidants, what – probably – represents the defence mechanism [27].

Additionally, there is substantial scientific evidence that the thyroid gland has perfectly developed a kind of autoregulation in terms of keeping redox balance under physiological conditions. For example, thyroglobulin, a specific thyroid glycoprotein, which provides sites for iodide organification, negatively regulates different components of thyroid hormone synthesis, those related to oxidative stress included (e.g. [28,29]).

Protective mechanisms against huge oxidative stress have been developed to protect not only the thyroid gland but also the whole organism. Specific anatomical feature of the thyroid gland, i.e. a monolayer of thyrocytes surrounding the thyroid colloid, does separate the colloid from the circulation, avoiding the leakage of ROS into the blood and thereafter to other tissues.

### Oxidative damage induced by Fenton reaction substrates

Fenton reaction substrates (Fe^2+^ or/and H_2_O_2_) were used in porcine thyroid to induce oxidative damage to membrane lipids (lipid peroxidation, LPO), to nuclear DNA and to mitochondrial DNA (mtDNA).

We found in our earlier *in vitro* study that H_2_O_2_ is not indispensable to induce LPO via Fenton reaction in porcine ovary homogenates, which means that external Fe^2+^ reacted with endogenous H_2_O_2_[13]. Therefore we expected that the use of only one Fenton reaction substrate would be sufficient to induce LPO via Fenton reaction also in the thyroid gland, especially that both, Fe^2+^/Fe^3+^ and H_2_O_2_ are required for thyroid hormone synthesis, thus being presumably in high concentrations in this gland. In opposite to our expectation, none of Fenton reaction substrates, used separately, increased LPO in the thyroid gland [30].

Expectedly when Fe^2+^ and H_2_O_2_ were used to-gether, LPO increased significantly, however without H_2_O_2_ concentration-dependent effect, but with clear Fe^2+^-dependent effect [30]. Thus, Fe^2+^ was found to be a stronger damaging factor to membrane lipids than H_2_O_2_, but it still required the presence of external H_2_O_2_ to reveal damaging effect. These unexpected results can be explained by the fact that the thyroid gland is specialized to compartmentalize H_2_O_2_, making it unavailable for any undesirable reactions. Such an explanation is supported by results mentioned above and showing that the increase of LPO occurring in the presence of both exogenous Fenton reaction substrates did not depend on the concentration of H_2_O_2_. In other words, the addition of small amounts of H_2_O_2_, resulting in its low concentrations such as 10^-5^ mM (the lowest concentration used in the above cited study), was sufficient, but still indispensable, to induce LPO in thyroid homogenates. It should be stressed that the lowest H_2_O_2_ concentration used in the study (10^-5^ mM) belongs to the range reached physiologically in cells – they vary from 0.001 μM to 15 μM [5,31], however other than thyroid cells. Thus, not compartmentalized H_2_O_2_ concentration in the thyroid is possibly much lower than in other tissues, but that should be experimentally proved.

Interestingly, when we induced LPO by Fenton reaction in porcine thyroid homogenates [12] (in this earlier study we did not check concentration-dependent effect of Fenton reaction substrates), melatonin (N-acetyl-5-methoxytryptamine), being perfectly documented as an effective antioxidant [11,32-34], prevented LPO with the lowest effective concentration of 0.25 mM. At the same time melatonin did not affect the basal LPO [12], which supports the statement that this indoleamine is an excellent antioxidant.

When nuclear and mtDNA were exposed to Fenton reaction substrates, they appeared to be more sensitive than membrane lipids, i.e. 8-oxo-7,8-dihydro-2^′^-deoxyguanosine (8-oxodG) concentration (DNA damage index) increased in the presence of only one Fenton reaction substrate [30,35]. Again, Fe^2+^ appeared to be more stronger damaging factor to both, nuclear DNA [30] and mtDNA [35], than was H_2_O_2_. The most important observation concerning oxidative damage to nuclear DNA and mtDNA is that the background level of 8-oxodG in mtDNA [35] was approximately 10 times higher than that one in nuclear DNA [30]. This disproportion between mtDNA and nuclear DNA is in agreement with results found in other tissues [36,37]. Concerning our observation on high mtDNA sensitivity to oxidative stress and the fact that thyroid oncocytic tumours are composed of cells filled – almost exclusively – with mitochondria characterized by molecular and enzymatic abnormalities, the following hypothesis can be formed. Whereas huge oxidative stress in the thyroid is hypothesized to play a crucial role in thyroid cancer, especially in papillary thyroid carcinoma, initiation [38], high oxidative damage to mtDNA in the thyroid allow to propose that oxidative processes substantially contribute to formation of thyroid tumors, with oxyphilic type of follicular thyroid carcinoma being of special significance [35].

In rat thyroid cell line (PCCl3), H_2_O_2_, used in concentrations 0.1-0.5 mM, caused a large number of DNA single-strand breaks and double-strand breaks. These data support the hypothesis that the generation of H_2_O_2_ in the thyroid could also play a role in mutagenesis, particularly in the case of antioxidant deficiency [39]. Additionally, these results [39] are in agreement with our findings [30,35] showing, that the use of only one Fenton reaction substrate, in this case of H_2_O_2_, is sufficient to induce oxidative damage to DNA.

### Oxidative damage induced by potassium bromate (KBrO_3_)

Potassium bromate (KBrO_3_) has been classified by International Agency for Research on Cancer (IARC) as a compound belonging to the group 2B of carcinogens (a possible human carcinogen) [40]. It has been demonstrated to cause, among others, follicular cell tumors in rat thyroid [41,42]. In our *in vivo* model single injection of KBrO_3_ (110 mg/kg b.w., *i.p*.) expectedly induced LPO in the rat thyroid gland [43]. That prooxidative effect of KBrO_3_ was completely prevented by melatonin (0.0645 mmol/kg b.w., *i.p*., twice daily, for 10 days), but also by indole-3-propionic acid (IPA) (0.0645 mmol/kg b.w., *i.p*., twice daily, for 10 days) – an indole substance with chemical structure similar to melatonin, and also by propylthiouracil (PTU) (0.025% solution in drinking water, for 10 days) – an antithyroid drug possessing certain antioxidative properties [43].

In *in vitro* study KBrO_3_ induced – in concentration-dependent manner (for concentrations of 5 mM and 10 mM) – LPO in porcine thyroid homogenates [43]. However, LPO caused by KBrO_3_ (5 mM) was not prevented by melatonin, and also by the IPA. Only PTU (in concentrations of 5.0, 7.5 and 10.0 mM) significantly reduced KBrO_3_-induced LPO in homogenates of porcine thyroids [43].

The explanation of this apparent discrepancy (between *in vivo* and *in vitro* study) may be as following. First, the lack of protective effect of potential antioxidants in the *in vitro* conditions does not exclude their ability to reveal such an effect in living organisms; this observation has been confirmed for melatonin and IPA in the above mentioned study [43]. Second, molecules, like melatonin and IPA, act as endogenous electron donors, primarily detoxifying ROS; this mechanism may be responsible for the high efficacy of antioxidative action of melatonin and IPA *in vivo*. Because these indoles do not possess the redox-active hydroxyl group at position 5 of the indole ring, they do not act as chain-breaking antioxidants and, therefore, they are either poorly protective or not effective *in vitro*[44].

It should be also stressed, that the protective effects of PTU against KBrO_3_-induced LPO in the thyroid *in vivo* and *in vitro* only allow proceeding into details of the antioxidative mechanism of PTU action, but they do not allow recommending PTU for protection specifically against oxidative damage and against thyroid cancer.

### Oxidative damage induced by nitrobenzene

Nitrobenzene is classified by IARC as possibly carcinogenic to humans (group 2B) [45]. It has been demonstrated, that nitrobenzene may induce thyroid follicular adenomas in mice, or both follicular thyroid adenomas and carcinomas in rats [46]. In our *in vitro* model nitrobenzene in concentrations of 7.5 and 10.0 mM induced LPO in porcine thyroid homogenates [Zasada and Karbownik-Lewińska, unpublished observations]. Nitrobenzene (7.5 mM) induced LPO was effectively prevented by melatonin, with its lowest effective concentration of 0.0001 mM. It should be stressed that this concentration of melatonin is only two orders of magnitude higher than physiological blood concentration in humans [Zasada and Karbownik-Lewińska, unpublished observations]. PTU also significantly reduced nitrobenzene-induced LPO, but only in its highest used concentration of 7.5 mM [Zasada and Karbownik-Lewińska, unpublished observations].

### Oxidative damage caused by components of somatotrophic axis

The increased incidence of cancer of different kind [47], the presence of goiter and thyroid cancer [48,49] in acromegalic patients and certain experimental evidence of growth hormone (GH) signalling effects on thyroid growth [50], and also potential cancerogenic properties of insulin-like growth factor I (IGF-I) justify to evaluate effects of somatotrophic axis components on oxidative damage to macromolecules, also in the thyroid.

In our *in vitro* study, GH, used in concentrations of 100, 10, 1.0, 0.01 μg/mL, and/or IGF-I, used in concentrations of 1000, 100, 10, 1.0, 0.1, 0.01, 0.001, 0.0001 μg/mL, did not change the basal LPO in porcine thyroid homogenates [51]. This effect was unexpected, but very desirable [51]. This finding may indicate that GH and IGF-I are very promising agents in terms of protection against oxidative damage under physiological conditions. At the same time, both GH and IGF-I significantly affected Fenton reaction-induced LPO. Namely, GH, in the lowest used concentrations (0.001 and 0.0001 μg/mL), prevented oxidative damage caused by Fenton reaction, thus revealing antioxidative effects; those effects of GH were the only protective effect against experimentally induced LPO, observed in this study. In other – higher (100, 10, 1.0, 0.1, 0.01 μg/mL) – used concentrations, we observed prooxidative effect of GH, i.e. GH enhanced Fenton reaction-induced LPO. In turn, IGF-I in all used concentrations enhanced Fenton reaction-induced LPO in porcine thyroid homogenates [51].

The fact that both GH and IGF-I may enhance oxidative damage initiated by other prooxidants does not support the idea that they may serve as protective factors under pathological conditions (other than GH deficiency).

### Oxidative damage caused by deficiency of antioxidative components

The thyroid gland is characterized by relatively high level of selenium [25,52]. Selenoproteins seem to be crucial for antioxidative protection in the thyroid, for thyroid hormone synthesis and for the global integrity of thyrocytes, as they are present in antioxidative enzymes such as GSH-Px, thioredoxin reductases, and also in deiodinases [25,52]. When murine thyroids were experimentally depleted of selenoproteins at gene level, expectedly was found the increased oxidative damage to membrane lipids [evaluated by the increased 4-hydroxynonenal (4-HNE) level], and to proteins (evaluated by increased 3-nitro-tyrosine level) [53]. Although oxidative damage to DNA was not evaluated in this study, it is expected that this molecule in the thyroid is also damaged under conditions of selenium deficiency. The above results clearly suggest that under physiological conditions selenoproteins protect thyrocytes from oxidative damage to macromolecules.

### Oxidative damage in thyroid cancer

The level of oxidative damage to macromolecules was found to be increased in cancerous thyroid tissue.

For example, in case of human thyroid diseases – non-toxic nodular goitre, carcinomas (follicular and papillary) and follicular adenoma – the highest level of LPO products was found in carcinomas; interestingly, it was also increased in follicular adenoma. Those changes were accompanied by increased activities of antioxidative enzymes, such as SOD, GSH-Px, and CAT, especially in case of thyroid carcinomas [54], and also in follicular thyroid adenomas and carcinomas [55].

In agreement with the above, increased levels of 8-oxodG and of 4-HNE were found not only in the cancerous tissues, such as follicular thyroid carcinoma and papillary thyroid carcinoma, but also in follicular thyroid adenoma [56]. Such findings [54-56] suggest that oxidative damage to macromolecules may be an early event of thyroid cancer that may affect disease progression.

Whereas the level of antioxidative defence increases in differentiated thyroid cancer, a decreased expression of mRNA encoding for CAT and SOD were found in anaplastic thyroid carcinoma, when compared to histopathologically unchanged thyroid tissue and to differentiated thyroid tumors [57]. Thus, in case of advanced stages of thyroid diseases, like e.g. anaplastic carcinoma, the defence mechanisms remain inactive or depressed.

## Conclusions

The increased oxidative damage to macromolecules in the thyroid occur in response to different exogenous prooxidants. The contribution of this oxidative damage to the development of thyroid diseases, cancer included, should be considered.

## Abbreviations

Asc^•^: Ascorbate radical; CAT: Catalase; DIT^•^: Diiodotyrosyl residue radical; DUOX: Dual oxidase; Fe^2+^: Ferrous ion; GH: Growth hormone; GSH: Glutathione; GSH-Px: Glutathione peroxidase; 4-HNE: 4-hydroxynonenal; H_2_O_2_: Hydrogen peroxide; I^•^: Iodine radical; I^+^: Iodinium ion; IARC: International Agency for Research on Cancer; IGF-I: Insulin-like growth factor I; [IO^-^ (IOH)]: Hypoiodous acid intermediate; IPA: Indole-3-propionic acid; KBrO_3_: Potassium bromate; LPO: Lipid peroxidation; mtDNA: Mitochondrial DNA; NADPH: Nicotinamide adenine dinucleotide phosphate; NO^•^: Nitric oxide; NOX: NADPH oxidase; 8-oxodG: 8-oxo-7,8-dihydro-2′-deoxyguanosine; ^•^OH: Hydroxyl radical; Prxs: Peroxiredoxins; PTU: Propylthiouracil; ROS: Reactive oxygen species; SOD: Superoxide dismutase; Tg-DIT^•^: Diiodotyrosyl residue radical in thyroglobulin; TPO: Thyroperoxidase; TSH: Thyrotropin; Tyr^•^: Tyrosine free radical.

## Competing interests

The Authors declare that there is no conflict of interest.

## Authors’ contributions

MKL wrote the manuscript and supervised preparation of the final version of the manuscript; AKB was involved in preparation of the manuscript. Both authors read and approved the final manuscript.
